# Influence of sevoflurane combined with propofol anesthesia on the anesthesia effect and adverse reactions in children with acute appendicitis

**DOI:** 10.1515/med-2025-1224

**Published:** 2025-12-06

**Authors:** Peng Zhang, Jun Zhang, Feng Chen

**Affiliations:** Department of Anesthesiology, Yantai Yeda Hospital, Yantai Economic and Technological Development Zone, Yantai, Shandong, 264006, China; Department of Anesthesiology, Yantai Yuhuangding Hospital, Yantai, Shandong, 264000, China; Department of Anesthesiology, Yantai Yuhuangding Hospital, No. 23, Huanghe Road, Yantai Economic and Technological Development Zone, Yantai, Shandong, 264000, China

**Keywords:** sevoflurane, propofol, combined anesthesia, acute appendicitis in children, surgical treatment, anesthesia effect, adverse reactions

## Abstract

**Objective:**

This study aimed to investigate the effect of sevoflurane combined with propofol anesthesia on the anesthesia effect and adverse reactions in children undergoing acute appendicitis surgery.

**Methods:**

A total of 104 children with acute appendicitis who underwent surgery were included and assigned into the observation group (sevoflurane combined with propofol anesthesia, 55 cases) and the control group (propofol anesthesia, 49 cases). The two groups were compared in terms of anesthesia effects, recovery quality, and the occurrence of adverse reactions, while observing changes in patients’ hemodynamics and stress levels during anesthesia.

**Results:**

Riker sedation and agitation scores were reduced in the observation group compared to the control group. The time to disappearance of pain and eyelash reflexes and the induction time of anesthesia were shortened, and heart rate and mean arterial pressure improved in the observation group. Cortisol, norepinephrine, and adrenocorticotropic hormone decreased in the observation group (*P* < 0.05). The occurrence of adverse reactions was not significantly different between the two groups (*P* > 0.05).

**Conclusion:**

The combination of sevoflurane and propofol anesthesia in children with acute appendicitis can enhance the anesthesia effect and recovery quality and stabilize the body’s hemodynamics and stress state.

## 1 Introduction

Appendicitis in children is a pediatric acute abdomen condition, marked by a hidden onset, a high chance of perforation, and a propensity to develop into diffuse peritonitis. Clinically, bacterial infection, appendix cavity obstruction, blood flow disorder, and neurological factors are believed to be main contributing factors [1]. At present, surgery is often applied to treat acute appendicitis in children. Anesthesia is the primary method of surgical analgesia and protects the respiratory and circulatory systems [2,3]. However, considering the young age of pediatrics, anesthesia sensitivity issues, and the many uncertainties in the anesthesia process [4], the development of a targeted anesthesia plan based on the characteristics of pediatrics is the key to facilitating a smooth surgical procedure.

Propofol, an intravenous anesthetic with an ultra-short duration of action, is advantageous due to its short half-life, lack of significant metabolic concerns post-infusion, and quick recovery following discontinuation. However, its analgesic effect is not good [5]. Sevoflurane is a widely used inhalation anesthetic in medical settings, known for its quick induction, simple anesthesia adjustment, stable anesthesia process, and minimal respiratory tract irritation [6,7]. Compound anesthesia has attracted much attention in clinical procedures in recent decades. It not only takes advantage of the synergistic effect between drugs, but also relatively reduces the amount of anesthetics used, thus reducing the risk of dose-related adverse effects and ensuring a more stable and anesthetic effect. A typical example of balanced anesthesia is the use of both intravenous and inhalation methods [8]. It maximizes the benefits of both inhalation and intravenous anesthesia, effectively managing surgery-induced cardiovascular reflexes without significant circulatory suppression, and better supports anesthesia maintenance [9]. Sevoflurane inhalation combined with intravenous isoproterenol anesthesia can achieve better anesthetic effects [10]. However, the effect of combined anesthesia with propofol on children is still inconclusive.

Therefore, the purpose of this study was to figure out the effect of sevoflurane combined with propofol on the anesthesia effect and adverse reactions in children with acute appendicitis surgery, and to offer a reference for the selection of anesthesia methods for children.

## 2 Materials and methods

### 2.1 Clinical data

From February 2019 to August 2021, a total of 104 children with acute appendicitis who underwent surgical treatment were included and assigned into the observation group (sevoflurane combined with propofol anesthesia, 55 cases) and the control group (propofol anesthesia, 49 cases). No significant difference was found in clinical data between the two groups (*P* > 0.05, Table 1).

**Table 1:** Comparison of clinical data between the two groups

Factors		Observation group (*n* = 55)	Control group (*n* = 49)	*χ* ^2^/*t*	*P*
Gender, *n* (%)	Male	31 (56.36)	26 (53.06)	0.114	0.736
	Female	24 (43.64)	23 (46.94)		
Age (years), mean ± SD		7.94 ± 1.61	7.75 ± 1.35	0.648	0.519
ASA classification, *n* (%)	Class I	21 (38.18)	22 (44.90)	0.482	0.488
	Class II	34 (61.82)	27 (55.10)		
Weight (kg), mean ± SD		25.17 ± 4.32	24.49 ± 4.51	0.785	0.434
BMI (kg/m^2^), mean ± SD		16.20 ± 2.16	15.82 ± 1.95		
Symptom duration (h), mean ± SD		2.13 ± 0.38	2.05 ± 0.34	1.126	0.263
Body temperature on admission (℃), mean ± SD		36.72 ± 0.31	36.65 ± 0.36	1.065	0.289
White blood cell count (*10^9^/L), mean ± SD		13.18 ± 2.04	12.89 ± 2.16	0.704	0.483
C-reactive protein (mg/L), mean ± SD		474.15 ± 41.47	480.28 ± 39.14	0.773	0.442
Procalcitonin (ng/L), mean ± SD		0.31 ± 0.05	0.29 ± 0.06	1.853	0.067

### 2.2 Inclusion and exclusion criteria

Inclusion criteria included: patients aged 2–12 years; all patients were in American Society of Anesthesiologists (ASA) rate class I–II; appendicitis was diagnosed by comprehensive examination of clinical signs, manifestation, and imaging; patient underwent laparoscopic appendectomy; and the family members of the patients signed the consent form.

After evaluation, 115 children were enrolled in the study and 11 patients were excluded: 2 patients with anemia (hemoglobin <110 g/L); 1 patient with long-term use of sedative drugs; 1 patient with a history of epilepsy; 2 patients with heart, liver, and kidney dysfunction; 1 patient with intracranial arteriovenous malformation; 3 patients with upper respiratory tract infection; and 1 patient was allergic to propofol or sevoflurane and other drugs used in this study.

### 2.3 Methods

All children were fasted for at least 6 h. Preoperative intravenous hydration and antibiotics (gentamicin plus clindamycin) were prescribed by the on-call emergency pediatrician and/or pediatric surgeon. Normal saline (0.9%) was used throughout the procedure. An anesthesia monitor (General Electric Company, Boston, MA, USA) was used to monitor the child’s vital signs.

To initiate anesthesia, 0.1 mg/kg of midazolam was administered (Jiangsu Enhua Pharmaceutical Co., Ltd), remifentanil at a dosage of 10 μg/kg (Jiangsu Enhua Pharmaceutical Co., Ltd) plus 0.1 mg/kg of cisatracurium (Zhejiang Xianju Pharmaceutical Co., Ltd). Post-induction, the procedure involved endotracheal intubation and the use of an anesthesia device (Yi’an anesthesia machine Aeon7200, Shanghai Hanfei Medical Device Co., Ltd) served as a means for mechanical air circulation. The measured tidal volume ranged between 8 and 10 mL/kg, with the breathing rate occurring 14–20 times per minute.

For anesthesia maintenance, the control group was infused with propofol (1.5 mg/kg/h; Guangdong Jiabao Pharmaceutical Co., Ltd) intravenously, and the observation group was infused with propofol intravenously and treated with sevoflurane by inhalation (2% MAC; Foton Gutian Pharmaceutical Co., Ltd).

During surgery, the bispectral index (one of several techniques used to monitor the depth of anesthesia [11] was maintained between 40 and 60%. In both groups, the patients were given continuous oxygen by mask with an oxygen flow rate of 1 L/min during the procedure.

All cases were operated by the same surgeon, and both anesthesia were turned off at the end of the procedure and neuromuscular blockade was reversed by slow intravenous injection of neostigmine (0.05 mg/kg) and atropine (0.02 mg/kg). When the patient met the criteria for extubation (return of gag reflex, facial expression, and purposeful movement), he or she was successfully extubated and transferred to the postanesthesia care unit.

### 2.4 Observation indicators

[1] Anesthesia effect: Riker sedation-agitation scale (SAS) [12] was evaluated before anesthesia induction, at the time of the eyelash reflex disappearing, at the beginning of the operation, 5 min after the operation began, 10 min after the operation began, and at the end of the operation. On a scale of 1 to 7, the higher the score, the worse the anesthetic effect [2]. Surgery-related indicators: the operation time, intraoperative blood loss, postoperative eating time, and postoperative ambulation time were compared between the two groups [3]. Indices related to anesthesia and recovery: the disappearance time of pain reflex and eyelash reflex, anesthesia-induced unconsciousness time, spontaneous breathing recovery time, consciousness recovery time, and tracheal extubation time were compared between the two groups. [4] Hemodynamics: pulse oxygen saturation (SPO_2_), heart rate (HR), and mean arterial pressure (MAP) of the two groups before anesthesia induction (T1), after surgical incision (T2), and at the time of extubation (T3) [5]. Stress indicators: radioimmunoassay was applied to detect cortisol (Cor), noradrenaline (NE), and adrenocorticotropic hormone (ACTH) at each time point (T1, T2, and T3).

### 2.5 Statistical analysis

The pilot study, which included ten children, utilized the technique for both groups, with five patients per group. The sample size was calculated considering a 15% change in MAP at T3. In order to achieve 90% test efficacy, 5% significance level, and 90% confidence interval, a minimum of 40 patients per group was required. The sample size of this study (55 patients in the observation group and 49 patients in the control group) met the statistical requirements. SPSS 22.0 software was employed to analyze the data. Enumeration data were shown as percent, and differences between groups were compared by Fisher’s exact test or *χ* ^2^ test. Measurement data were illustrated as mean ± standard deviation (SD). Normality of continuous variables was checked using Shapiro–Wilk test, and differences between the groups were compared by *t*-test. Changes in hemodynamic parameters and stress markers over time were analyzed using repeated-measures ANOVA. *P* < 0.05 emphasized significant statistical difference.


**Informed consent:** Informed consent was signed by the guardian of every subject.
**Ethical approval:** This study has been approved by the ethics committee of Yantai Yeda Hospital on date: 2018.06.10 (Ethics approval No. [YD2018-037]).

## 3 Results

### 3.1 Comparison of anesthesia effects

**Figure 1:**
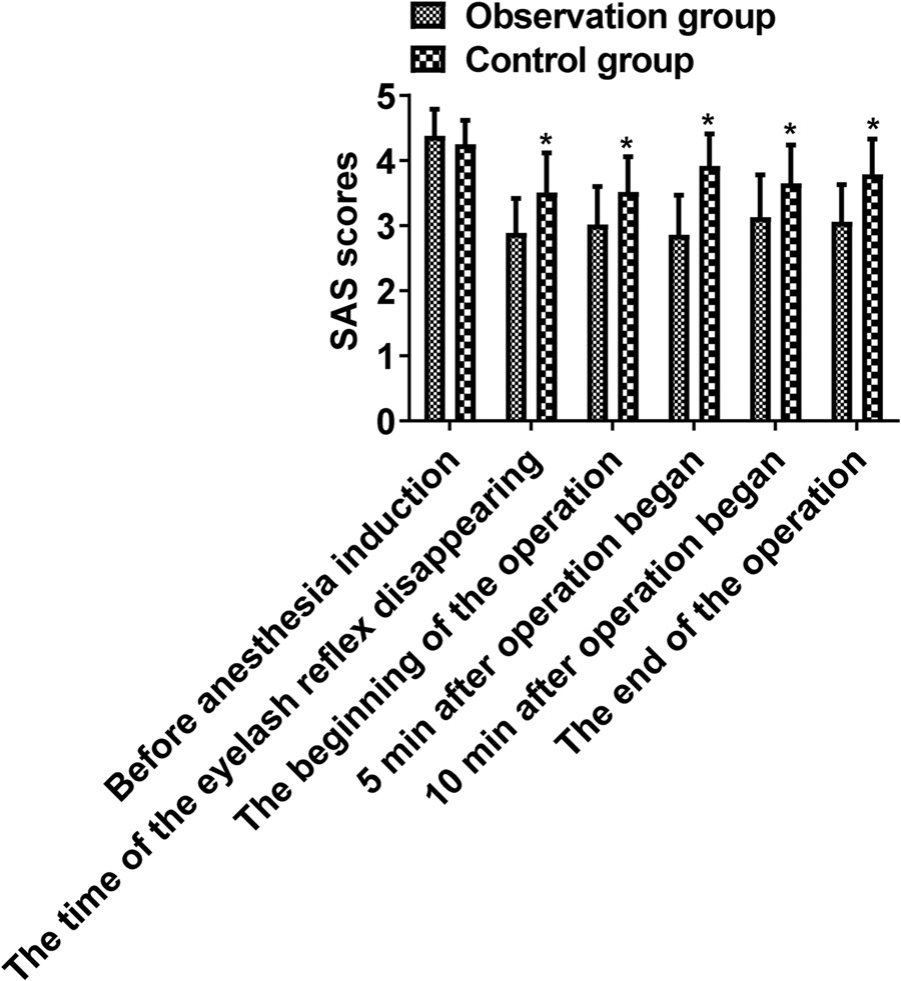
Comparison of SAS scores between the two groups at different time periods. Comparison of two groups, *P < 0.05.

The SAS scores of the observation group at the time of the eyelash reflex disappearing (2.89 ± 0.53 vs 3.51 ± 0.61), at the beginning of the operation (3.02 ± 0.58 vs 3.52 ± 0.54), 5 min (2.86 ± 0.61 vs 3.92 ± 0.49) or 10 min after operation began (3.13 ± 0.65 vs 3.65 ± 0.59), and at the end of the operation (3.06 ± 0.57 vs 3.79 ± 0.54) were lower compared with the control group (*P* < 0.05, Figure 1 and Table 2). These results suggest that combined anesthesia optimizes intraoperative sedation and reduces child agitation.

**Table 2:** Comparison of anesthesia effects between the two groups

SAS scores at different time points	Observation group (*n* = 55)	Control group (*n* = 49)	*t*	*P*
Before anesthesia induction, mean ± SD	4.38 ± 0.41	4.25± 0.37	1.690	0.094
The time of the eyelash reflex disappearing, mean ± SD	2.89 ± 0.53	3.51 ± 0.61	5.546	<0.001
The beginning of the operation, mean ± SD	3.02 ± 0.58	3.52 ± 0.54	4.533	<0.001
5 min after operation began, mean ± SD	2.86 ± 0.61	3.92 ± 0.49	9.692	<0.001
10 min after operation began, mean ± SD	3.13 ± 0.65	3.65 ± 0.59	4.252	<0.001
The end of the operation, mean ± SD	3.06 ± 0.57	3.79 ± 0.54	6.683	<0.001

### 3.2 Comparison of surgery-related indicators

**Figure 2:**
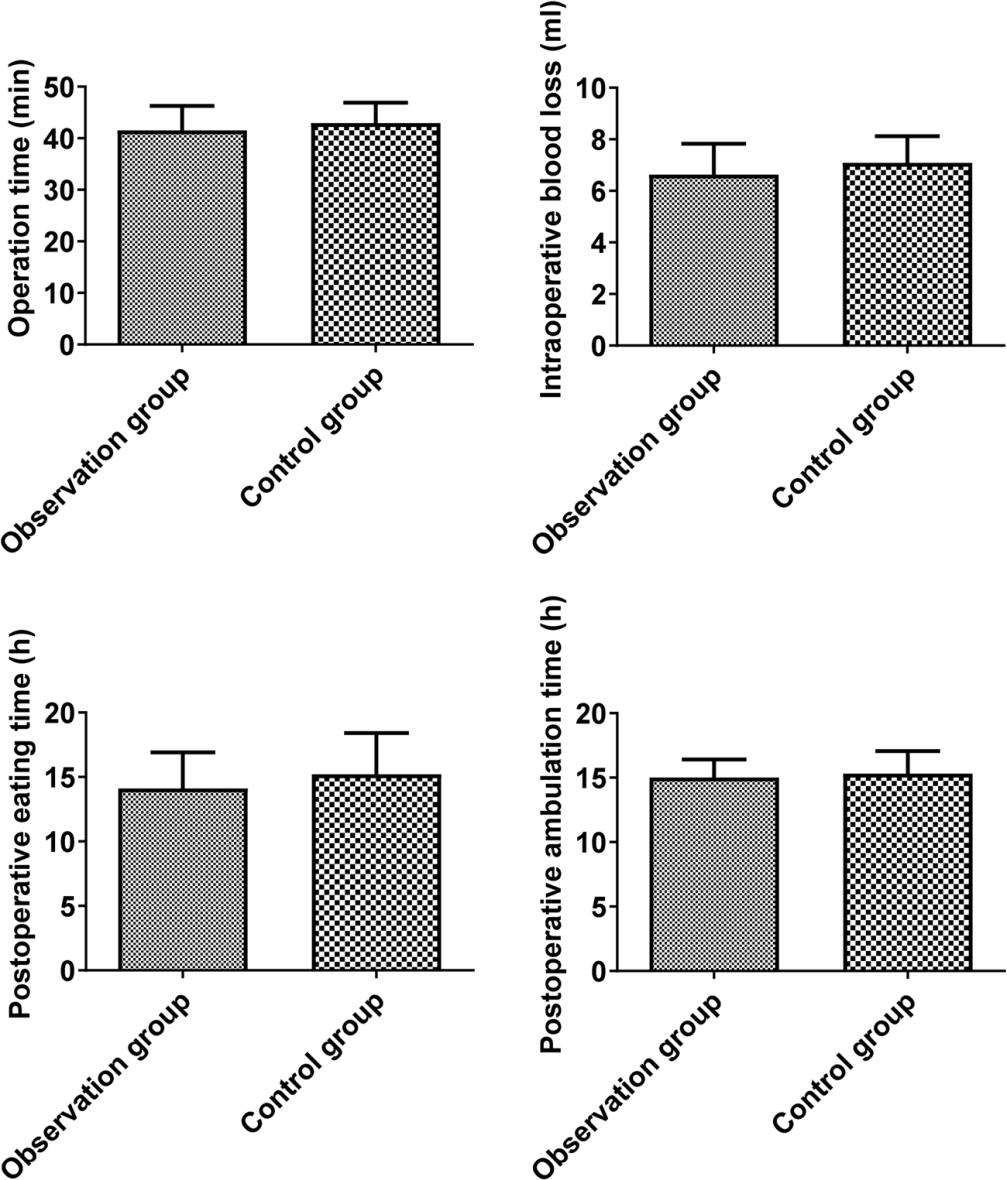
Comparison of operation-linked indicators between the two groups.

No significant difference was found in operation time (41.51 ± 4.75 vs 42.92 ± 3.97), intraoperative blood loss (6.63 ± 1.20 vs 7.09 ± 1.04), postoperative eating time (14.10 ± 2.80 vs 15.22 ± 3.20), and postoperative ambulation time (15.01 ± 1.40 vs 15.31 ± 1.76) between the two groups (*P* > 0.05, Figure 2).

### 3.3 Comparison of anesthesia-related indicators

**Figure 3:**
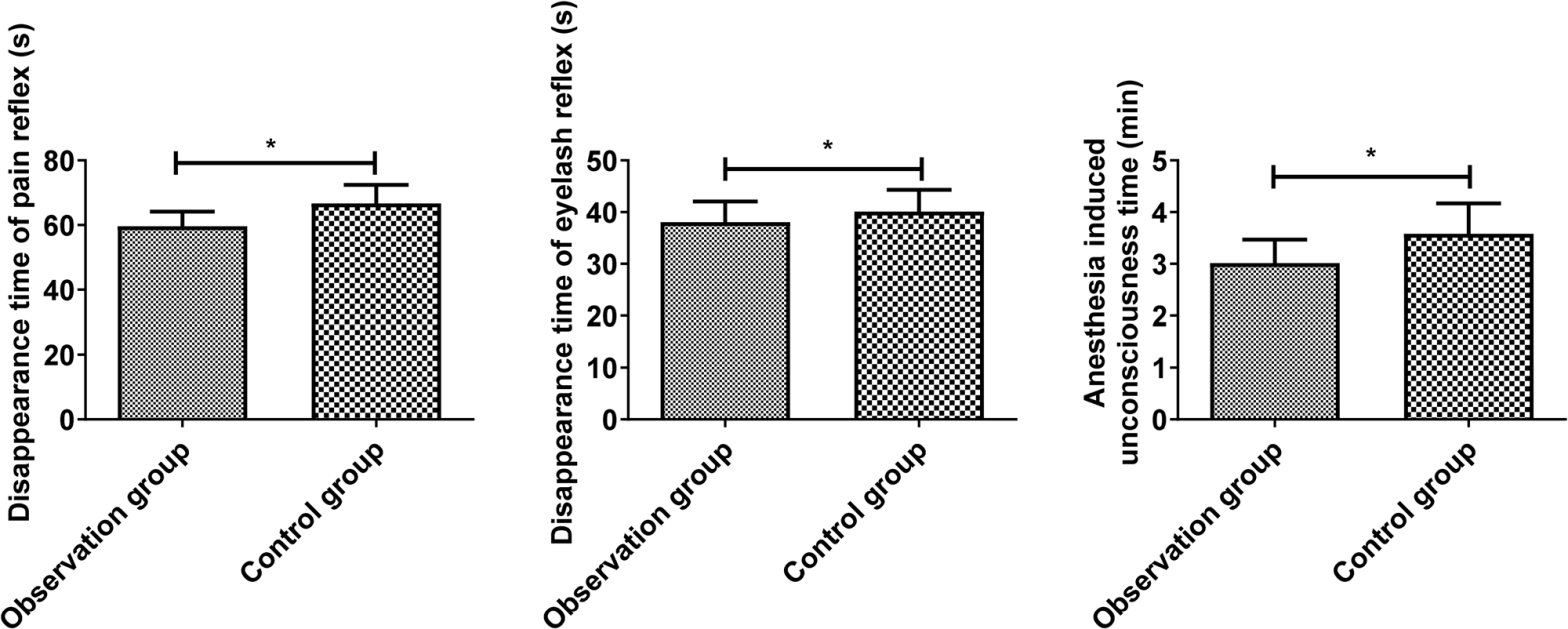
Comparison of anesthesia-linked indicators between the two groups. Comparison of two groups, *P < 0.05.

The disappearance time of pain reflex (59.64 ± 4.49 vs 66.67 ± 5.76) and eyelash reflex (38.07 ± 3.99 vs 40.10 ± 4.22) and anesthesia-induced unconsciousness time (3.01 ± 0.46 vs 3.58 ± 0.59) in the observation group were shorter compared with the control group (*P* < 0.05, Figure 3). These results suggest that combined anesthesia has a better anesthetic outcome.

### 3.4 Comparison of recovery quality

**Figure 4:**
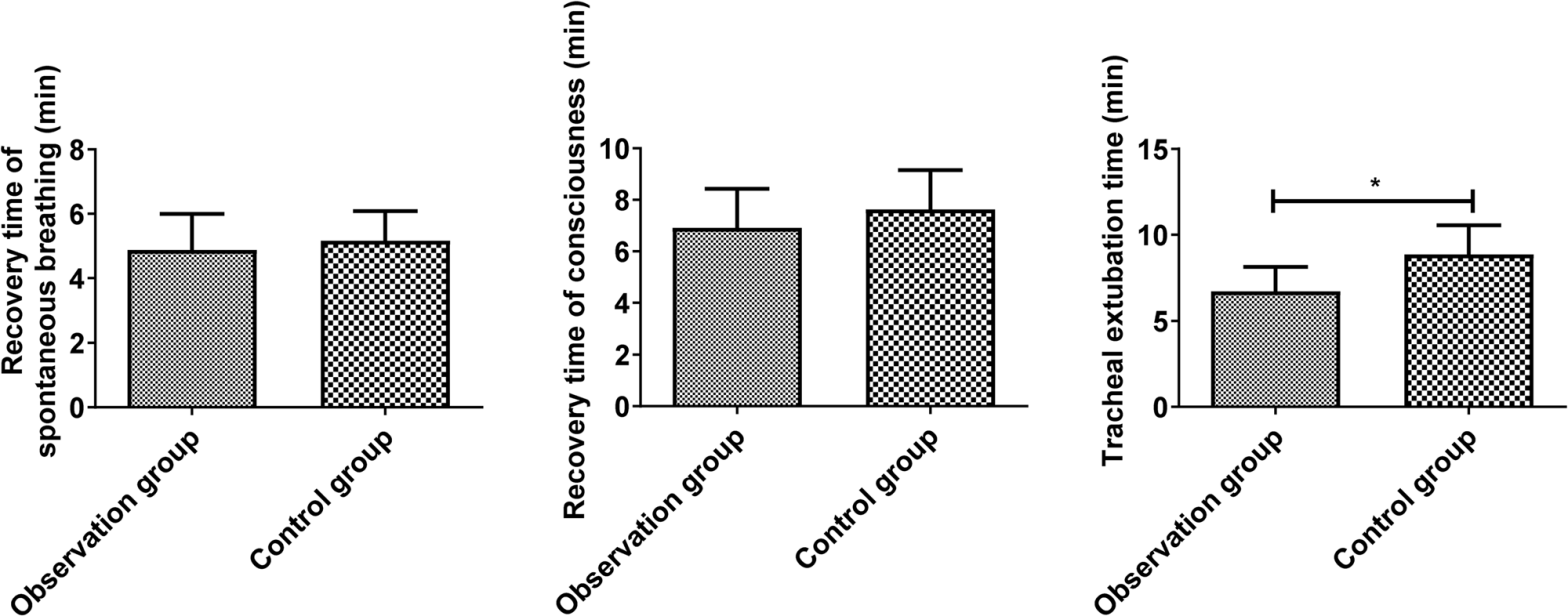
Comparison of wake-up quality of the two groups. Comparison of two groups, *P < 0.05.

No significant difference was detected in the recovery time of spontaneous breathing (4.88 ± 1.12 vs 5.16 ± 0.92) and consciousness (6.91 ± 1.52 vs 7.63 ± 1.53) between the two groups (*P* > 0.05). The tracheal extubation time (6.71 ± 1.42 vs 8.86 ± 1.69) in the observation was shorter compared with the control group (*P* < 0.05, Figure 4).

### 3.5 Comparison of intraoperative hemodynamics

**Figure 5:**
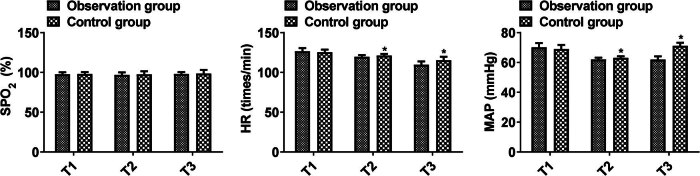
Comparison of intraoperative hemodynamics between the two groups. Comparison of two groups, *P < 0.05; T1: before anesthesia, T2: after surgical incision, T3: during extubation.

No significant difference was shown in SPO_2_, HR, and MAP at T1 (SPO_2_, 97.68 ± 2.45 vs 98.07 ± 2.38; HR, 126.58 ± 3.95 vs 125.39 ± 3.24; MAP, 70.19 ± 2.85 vs 69.05 ± 2.71) between the two group (*P* > 0.05). No significant difference was detected in SPO_2_ at T2 (96.92 ± 3.15 vs 97.61 ± 3.79) and T3 (98.05 ± 2.42 vs 98.53 ± 4.64) between the observation and the control groups (*P* > 0.05). HR and MAP at T2 (HR, 119.68 ± 2.09 vs 121.14 ± 2.18; MAP, 62.04 ± 1.21 vs 63.13 ± 1.03) and T3 (HR, 109.68 ± 4.12 vs 115.37 ± 4.35; MAP, 61.94 ± 2.13 vs 71.16 ± 2.04) in the observation were higher than the control (*P* < 0.05, Figure 5). These results suggest that combined anesthesia suppresses the hemodynamic response to injurious stimuli.

### 3.6 Comparison of stress indicators

**Figure 6:**
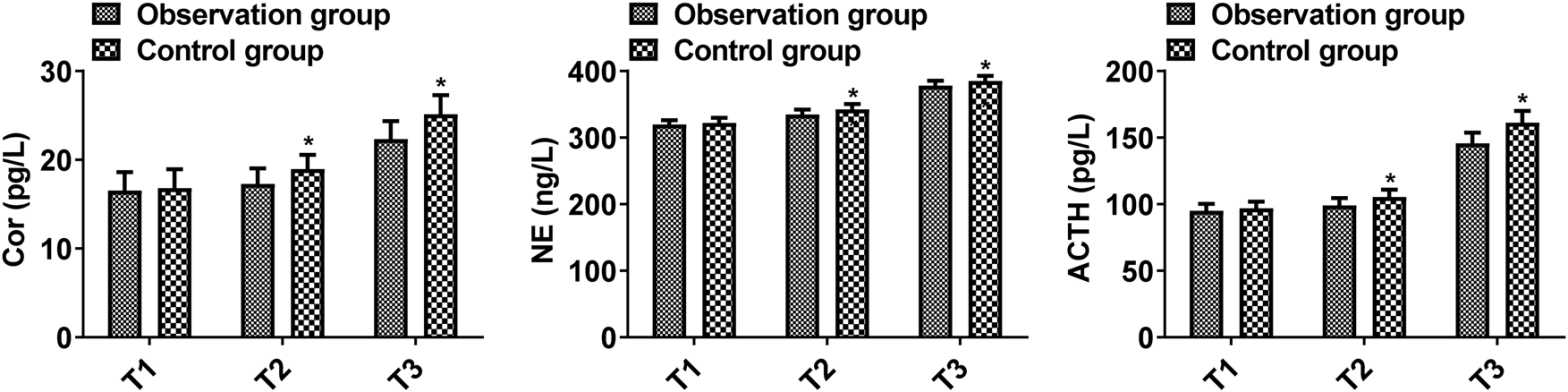
Comparison of stress indicators between the two groups. Comparison of two groups, *P < 0.05; T1: before anesthesia, T2: after surgical incision, T3: during extubation.

No significant difference was presented in Cor, NE, and ACTH at T1 (Cor, 16.53 ± 2.09 vs 16.81 ± 2.13; NE, 319.85 ± 6.31 vs 322.07 ± 7.68; ACTH, 95.16 ± 5.12 vs 96.85 ± 5.05) between the two groups (*P* > 0.05); Cor, NE, and ACTH at T2 (Cor, 17.28 ± 1.75 vs 18.96 ± 1.59; NE, 334.68 ± 7.65 vs 342.45 ± 7.92; ACTH, 99.07 ± 5.47 vs 105.34 ± 5.63) and T3 (Cor, 22.34 ± 2.03 vs 25.13 ± 2.15; NE, 378.05 ± 7.31 vs 385.16 ± 7.56; ACTH, 145.68 ± 8.12 vs 161.19 ± 8.96) in the observation were lower compared with the control group (*P* < 0.05, Figure 6). These results suggest that combined anesthesia can reduce the systemic stress response triggered by surgical trauma.

### 3.7 Comparison of the incidence of anesthesia-linked adverse reactions

Adverse reaction rates were not significantly different between the groups, being 7.27 and 6.12% (*P* > 0.05, Table 3). These results indicate that combined anesthesia did not increase the risk of nausea and vomiting, drowsiness, or respiratory depression and has a high safety profile.

**Table 3:** Comparison of the incidence of anesthesia-related adverse reactions between the two groups

Adverse reactions	Observation group (*n* = 55)	Control group (*n* = 49)	*χ* ^*2*^	*P*
Nausea and vomiting, *n* (%)	3 (5.45)	1 (2.04)		
Somnolence, *n* (%)	1 (1.82)	1 (2.04)		
Respiratory depression, *n* (%)	0 (0.00)	1 (2.04)		
Total, *n* (%)	4 (7.27)	3 (6.12)	0.055	0.815

## 4 Discussion

Laparoscopic appendectomy is often applied in clinical treatment of acute appendicitis. Since children have low tolerance, limited cooperation, and slower drug metabolism and excretion, suitable anesthetic drugs should be chosen during surgery to minimize stress response. Propofol is an intravenous anesthetic drug that has been widely used in the induction and maintenance of general anesthesia because of its non-accumulation and short recovery time [13,14]. However, previous studies suggest that the anesthetic effect of propofol alone is insufficient, making the combination with sevoflurane necessary [15]. Sevoflurane is an inhaled anesthetic that offers benefits such as lowering the need for muscle relaxants, quick recovery, effective control, and no respiratory tract irritation. Inhaled through the alveoli, it enters the blood, impacts the central nervous system, and is discharged through the respiratory tract, with a minor amount metabolized into fluoride [16,17]. In this study, sevoflurane was combined with propofol for surgical anesthesia in children with acute appendicitis. The results clarified that the SAS score of the observation group was reduced at the time of the eyelash reflex disappearing, at the beginning of the operation, 5 or 10 min after the operation began, and at the end of the operation. The disappearance time of the pain reflex and eyelash reflex and anesthesia-induced unconsciousness time were shorter in the observation group, indicating that combined anesthesia has better anesthesia effect, which is consistent with a relevant report [18]. Sevoflurane produces few metabolites and does not build up during anesthesia. It mildly depresses respiration in children, potentially shortening the time for spontaneous breathing recovery [19,20]. This research showed that using sevoflurane with propofol did not impact the recovery of spontaneous breathing and consciousness, possibly due to the limited sample size, suggesting a need for further investigation.

Surgery and anesthesia can trigger a stress response, impacting postoperative recovery. Effective anesthesia is essential for enhancing patients’ postoperative stress state [21]. Propofol reduces the stress response by activating GABA receptors, facilitating intracellular chloride ion conductance, decreasing peripheral resistance and venous tone, and directly inhibiting cardiovascular nerve reflexes. However, because of the suboptimal anesthetic effect of propofol, it may increase the stress response to surgical stimuli in humans [22,23]. This study found that Cor, NE, and ACTH at T2 and T3 in the observation group were lower compared with the control group, indicating that sevoflurane combined with propofol anesthesia can reduce the stress response of patients during acute appendicitis surgery. These data align with prior mechanistic studies demonstrating synergistic interactions between propofol and sevoflurane at hypothalamic receptors. Specifically, their combined action suppresses nociceptive signal transduction through dual modulation of glutamatergic and GABAergic pathways, ultimately inhibiting hypothalamic–pituitary–adrenal (HPA) axis hyperactivity and stress hormone secretion [24].

In children with acute appendicitis surgery, perioperative emotional changes, drug stimulation, and invasive operations can lead to large fluctuations in intraoperative hemodynamics. Research has indicated that external factors like surgery can trigger hemodynamic changes, leading to abnormal fluctuations and activating the HPA cortex axis, thereby intensifying the body’s stress response [25,26]. In this study, it was found that combined anesthesia can improve the anesthetic effect and reduce the body’s stress reaction, suggesting that combined anesthesia helps to maintain hemodynamic stability. This study showed that the HR and MAP of the observation group were better than those of the control group at two time points, T2 and T3, suggesting that combined anesthesia is more conducive to maintaining intraoperative hemodynamic stability, mainly because sevoflurane inhalation anesthesia is less irritating to the airway and stabilizes the blood circulation [27]. This study found the time of tracheal extubation in the observation group was shorter than the control group, indicating that intravenous propofol anesthesia combined with sevoflurane inhalation anesthesia was helpful for postoperative extubation. This is mainly because sevoflurane anesthesia can inhibit the cardiovascular stress response caused by tracheal intubation, maintain a stable heart rate, inhibit myocardial excitability, reduce histamine release, lower blood pressure, and thus reduce the difficulty of postoperative extubation [28]. Propofol is similar in chemical structure to a phenol-based free radical scavenger and has a latent protective effect on the kidneys. Its application in general anesthesia can effectively reduce the incidence of adverse reactions of anesthesia [29]. This study found that sevoflurane and propofol did not increase the risk of adverse reactions, pointing to the high safety of using both anesthetics together.

However, this study has limitations. First, the sample size was small and secondary outcomes (e.g., adverse events) may have insufficient statistical validity. Second, postoperative pain scores and long-term neurocognitive function were not assessed. Future more in-depth analyses based on these points should be conducted to confirm or even improve the current findings.

In conclusion, the use of sevoflurane alongside propofol anesthesia in laparoscopic procedures for acute appendicitis in children enhances anesthesia quality and maintains stable hemodynamics and stress conditions. Also, this regimen is considered safe and effective.
